# Nineteenth Annual Meeting of the Ohio State Dental Society

**Published:** 1885-01

**Authors:** E. G. Betty


### Proceedings and Discussions of the Nineteenth Annual Meeting of the Ohio State Dental Society.

REPORTED BY E. G. BETTY, D.D.S.

The nineteenth annual meeting of this Society was called to order by Chairman Berry, in the Board of Trade rooms, at Columbus, O., Oct. 29th, 1884, at 10:30 A. M. Prayer was offered by the Rev. Mr. Lincoln. The roll was then called and dues paid. Dr. Robinson said that the Society had permission to use the rooms, provided it adjourned each day at 11 A. M.; this was agreed to. The Chairman of the Executive Committee was empowered to employ a stenographic reporter. Adjourned till 2 o'clock P. M.

FIRST DAY—AFTERNOON SESSION.

The meeting was called to order by President Berry, at 2:15. The treasurer, Dr. G. W. Keely read the names of the members resent who had paid their dues, also announcing that those who were in arrears two years, and had not paid up, could not participate in the proceedings until they had done so. The Secretary read the minutes of the morning session, and no objection being made they stood approved. Dr. Todd, in behalf of the Membership Committee, proposed the following gentlemen for membership: L. J. Mitchell, Delaware, graduate of Ann Arbor; B. R. Tabor, of Bowling Green, Wood county, graduate of the Philadelphia College; and A. O. Howe, of Washington C. H., a graduate of the Ohio Dental College, all of whom were elected. A communication was read from Dr. Alex Campbell, of Laronas Ferry, British Columbia, in which he asked for information as to his becoming a member; on motion, the letter was referred to the Committee on Membership. Dr. C. H. Land, of Detroit, Mich., was present as a visitor, and was accorded the privileges of the floor. Dr. Robinson, of the Executive Committee, made his report concerning the procurement of rooms for the Society to meet in, and the employment of a stenographic reporter; the report was accepted and adopted. Dr. James raised the question as to whom belonged the papers that were read before the Society, or at least he wanted to know who was the legal custodian. Dr. J. H. Warner said the custom was to turn them over to the Publication Committee and the Secretary. Dr. Harrison wished to have the Committee on Voluntary Essays instructed to take charge of papers, subject to the order of the Publication Committee.

*Dr. Harroun.* It is not necessary to do that, as it is already their duty.

Dr. Harrison then read his resolution, which he had changed.

(It appears that his remarks above were in the nature of a resolution.)

*Dr. Horton.* Better make it a by-law.

*Dr. Rehwinkel.* I don't understand what all this talk is about, but I do know that everything brought before the Society becomes its property. Some one has just referred to a resolution I offered in 1878; the exact circumstances under which that resolution was made I don't recollect, except that there was a great deal of squabbling about it. A member who reads a paper before the Society, gives up all right and title to it, and if it is not called in, it is by reason of neglect upon the part of the proper officers, whose business it is to look after the interests of the Society, in all ways.

*Dr. Horton.* A resolution is not a law, and can not be so construed.

*Dr. J. H. Warner* thought that reflections were being made upon the Secretary, judging from some of the remarks that had been made. He then went on to detail the story of the essays of last year finding their way into the office of the "*Independent Practitioner*," contrary to the rules of the Society.

*Dr. J. Taft.* This talk is a waste of time, is not pertinent, and I therefore move that it "be laid upon the table." Carried. Dr. Horton offered to read a report from the Committee on Dental Law, he being the chairman of that committee. This was objected to by a member, but Dr. Horton was requested to read his paper. The report was prefaced with some specifications as to who were members of the special committee of five on Dental Law, and how trouble had resulted. Owing to the distance the several members live apart, I have therefore to report that there has been no meeting of the members of your special committee on Dental Law.

*Dr. Warner.* I move the report be laid on the table.

Lost for want of a second.

*Dr. Taft* suggested that some definite time be set to consider the dental law, and moved that it be made a special order of business for Thursday at 10 o'clock A. M. Carried.

*Dr. Keely* moved that a committee of three be appointed by the President to confer with the Publication Committee, and sift the matter of the disappearance of the essays of the last meeting, and that Dr. Horton be present. Carried.

The Chair then appointed Drs. H. A. Smith, Emminger, and Hunter, on that committee.

Unfinished business was now in order.

*Dr. Taft.* Last year a suspension was placed upon two members of the Society, and I move that the matter be now taken up and suitable action taken upon it. Seconded.

*Dr. Sillito.* Dr. James' resolution of last year provided that the suspension continue till the last day of this meeting.

*Dr. Taft.* I had forgotten that, but it should be brought up now in order that it may be disposed of.

*Dr. Watt.* Dr. James' resolution can be rescinded.

*Dr. James.* I do not remember why my resolution named the last day of this meeting.

*Dr. Rehwinkel.* There may be new charges to make.

*Dr. Taft.* That is an additional reason why it should be taken up now.

The resolution of Dr. James was, on motion, rescinded.

*Dr. Taft.* I now offer my resolution.

*Dr. Horton* moved to make the subject a special order of business for the evening session. No second.

*Dr. Hunter.* It seems that for the past few years there has been continual bickering and quarreling among members with the result of a lessening attendance. It looks as if we had resolved ourselves into a mutual damnation society. Let us have the thing out now, and be done with it.

Dr. Taft's motion was then carried.

*Dr. Taft.* In order to bring the matter before the Society, I move that the suspension placed upon Drs. R. G. Warner and Gares, last year, be removed. Seconded.

*Dr. Rehwinkel.* From the Committee on Ethics, I call upon those gentlemen to make a satisfactory explanation in accordance with the provisions of Dr. James' resolution, and if they are ready to apologize, and hereafter comply with the code and rules of the Society.

*Dr. R. G. Warner.* I refuse to exhibit myself by being catechised before this body, as that can be done before the Committee on Ethics.

*Dr. Taft.* In view of Dr. Warner's objections, I move that the committee and he confer together now, and afterwards report the result to the Society.

*Dr. Rehwinkel.* Then re-read Dr. James' resolution. As they have refused compliance, I now move that they be expelled; seconded.

*Dr. James.* The gentleman have not come here with a penitent spirit by any means and are defiant; neither have they complied with the provisions of the agreement that was made when the suspension was put upon them as the following advertisement in a newspaper, published subsequently to the adjournment of the last meeting sufficiently proves.

The doctor then read another inflammatory advertisement embodying the criticisms of the daily press upon the propriety vs. the impropriety of professional gentlemen using the daily papers for the purpose of making their business known to the public.

*Dr. Horton.* That has nothing to do with the matter for which the gentlemen are under suspension.

*Chair.* The chair rules that Dr. James is in order and has the floor.

*Dr. Horton.* Then I appeal to the meeting.

This created a stir and was productive of many remarks, mostly in favor of Dr. James, and when the excitement was partially subsided, Dr. Horton's appeal was lost by an overwhelming vote.

*Dr. James.* As these men have been placed on probation by the Society, it has a right to know what they have done during that period. He then proceeded to finish the reading of their advertisements.

*Dr. Hunter.* I imagine that Dr. Warner did not fully understand the question put to him by Dr. Rehwinkel.

After a long tedious debate, Dr. Horton's motion to refer the subject back to the Committee on Ethics was lost.

The previous question on Dr. Rehwinkel's motion was then called and unanimously carried.

Dr. Horton called for a poll of the house, and out of a total of 22 votes there were 20 ayes and 2 nays. The vote on the expulsion of Dr. Gares was then announced, and he also was expelled, ayes, 14; nays, 7.

The Society then adjourned till evening.

FIRST DAY—NIGHT SESSION.

The session was called to order by President Berry at half-past seven; the minutes of the afternoon session were read, and there being no objection offered they stood approved as read. Drs. Emminger, Callahan, and Harrison were appointed by the Chair as a Committee on Voluntary Essays, as there were none of the regular members of that committee present. The Secretary read a letter from Dr. Wm. Mitchell, formerly of Ohio, who had since removed to London, England. The Secretary moved that the gentleman be elected an honorary member; carried.

Dr. James brought up the resolution from last year's meeting amending Article 2, Sectien 6, proposing to add the words "suspension and reprimand" to the word "expulsion." The amendment was carried. Another proposition to amend Section 7 of the By-Laws by striking out the words "To the next regular meeting of," and to substitute "report their decision immediately thereafter," was on motion carried. Dr. Horton called for the ayes and nays: the result was ayes, 13; nays, 4.

Dr. Callahan, of the Committee on Voluntary Essays, reported that he had a paper from Dr. E. G. Templeton, entitled "Prosthetic or Artificial *alias* Mechanical Dentistry," being relevant to the first subject on the programme, "Improvement in Artificial Dentures." *

At the conclusion of the reading of Dr. Templeton's paper, Dr. Taft read one on Improvement in Artificial Dentures which appeared in the last issue of the REGISTER. Dr. Harroun stated that Dr. Land, of Detroit, Mich., was present, who, being a spe-

* (We made a synopsis of the paper as Dr. Callahan read it, but will not insert it here, as the REGISTER, by action of the Society, has possession of the paper and published it in the December No.—REP.)

cialist in mechanical dentistry, would undoubtedly give the Society the benefit of his experience, and invited him to speak.

*Dr. Land.* Mr. President, I desire to thank the Society for the opportunity to say something at this meeting. When at home, on looking over the DENTAL REGISTER, I saw that there would be a discussion on Mechanical Dentistry, this brought me here with the hope of being able to learn something and perhaps say a word.

I think the whole fraternity is interested in the subject under discussion, and I will say that no man is a true dentist unless he is a whole dentist, we must neglect no part of the work on account of its having been degraded. The man who says I don't want to take hold of this or that sort of a job simply because it won't pay is more to blame than the man who advertises teeth at $5.00. Cheap dentistry has a track of its own and can justly be termed the enemy of art. Why is it cheap? Because it is the chromo branch of our profession; and why? Because the manufacturer has done what the dentist ought to do—has made teeth so perfect and cheap that the dentist cannot compete with him. The dentist receives teeth in sections of two and three, then he has only to put them on a piece of rubber and they are done. Now I don't care how perfect a workman a man may be, you set him down on section teeth and he is robbed of his art. If it is a fact that we cannot make a better set of teeth than the six dollar men, then we must submit to the competition and acknowledge that we are not better than chromo dentists.

I have a few specimens to show and speak of in which I am interested financially, although not to any great extent. However, I do not wish to take up the time of this Association by placing myself in the position of an advertiser.

*Dr. Taft.* Dr. Land can say all he wants to here on this subject, we want to know about these things and shall not look upon anything he may say in the light of an advertisement.

*Dr. Land.* I thank you. Well, I have perfected a new class of teeth having three pins, they are especially designed for continuous gum work and sections of six to be atttached to full sets of rubber (exhibits specimens of six front teeth which were to be completed by adding the bicuspid and molars of the ordinary gum sections, and thus make it possible to complete a continuous gum front for rubber work) that had proved to be entirely practical, durable, and easy to repair in case of accident, the entire cost not to exceed $4.50.

In addition to providing new teeth I have found it necessary to study the conditions of the mouth in each case, and have noted down a few facts in order to show the progress made to produce something better in several directions. 1. I found it necessary to study the mouth scientifically until each case became so familiar as to enable me to estimate with the utmost precision the amount of retaining force presented in each case, according to the angles and various conditions. The first step is to provide a proper system of relief governed by the conditions. 2. To provide a means of utilizing the pressure of the atmosphere for the temporary purpose of establishing an adaptation. 3. To provide a means that will have a tendency to retain moisture between the denture and the mouth, thus by capillary adhesion which represents a retaining force of about two pounds to the square inch, the denture is permanently maintained, while the exhausting power of the tongue is not more than two ounces to the square inch. It is a common error to suppose that we have fourteen pounds pressure to the square inch. Even if we could secure one pound to the square inch it would draw the mouth down so as to be painful and put it out of shape. When the mouth is simply relieved by creating a slight space covering four-fifths of the palatine surface it will soon conform to it, and in conjuction with the fluids of the mouth an equilibrium will be established. A dry, rigid mouth is the most difficult to fit: in this case you have a rigid denture and a rigid mouth with very little moisture. It would be much better to have a rigid mouth and a flexible denture, or the reverse, flexible mouth and a rigid denture. A piece of chamois skin placed between two rigid substances, will make a perfect adaptation and would be proper to use; however, when placed in the mouth it becomes foul and unclean, therefore not practical.

What we want is some indestructible substance that will have a tendency to support a small amount of moisture between the denture and the mouth. I have brought some models with me that were puzzles to many, all of which have been fitted with practical sets of continuous gum work showing the systems of reliefs adopted in order to reach the maximum adaptation.

(The doctor here exhibits several specimens of mouths taken in plaster, and discusses the relative conditions of the same, after which he proceeds as follows: )

Now there is an immense amount of work to be done by all classes of dentists, and I think we should be more charitable to the six dollar men; they certainly are a blessing to the poor. The people who generally employ them have very little appreciation for art; they are seeking for something practical and cheap, and they have as much right to receive it as those who are seeking for art and are willing to pay for it. I think every dentist who is an artist will always find a market—their work will be appreciated, and compensation sure to follow—and I am sure that there is as much, if not more, room in this world for such men than the others. I have many patients who can not pay for continuous gum work, $60 to $70, but can pay $30, therefore have found it necessary to give them something better than the chromo work and not so expensive as continuous gum; the combination, continuous gum and rubber work fills this requirement. As to dividing the prosthetic from the artificial methods of restoring the teeth, I don't think we will live to see the day when that can be done.

The great majority of men say, in regard to the cheap work, I don't do anything of that kind, operating pays so much better. It is unwise to turn away a patient simply because he or she wants a cheap set of rubber teeth. Every dentist should practice this class of work himself, unless he can delegate it to some competent person. We ought to do this, at least until we offer a better field of education for the young men, and show them where these things can be made useful, otherwise, give up some of your best patients to chromo dentists. Another point: there is a tendency to run down rubber work. It is not as good as metal—this is a fact. The former is more apt to make the mouth sore and tender; however I have seen very sore mouths under metal, caused from want of friction. Perhaps rubber work, owing to its non-conducting qualities, is a little worse, therefore it should not be condemned too much. Rubber will make a rigid mouth soft, therefore a better fit is secured than with metal. I was showing here, when exhibiting the specimens, a case where metal work was a complete failure. When I find a very dry, rigid, flat mouth, I know there is a very little retaining force. For this I want rubber, because it brings about the very condition that is needed; it softens the mouth, is lighter, and easy to adapt. Of course, metal plates can be adapted the same as rubber, but if I wanted to be very successful in this case, I would use rubber. In working continuous gum the gas furnace is destined to play an important part, it will certainly supersede the coal and coke furnaces. The advantage of being able to bake a set of teeth in from ten to twenty minutes, always certain, no dirt, no handling of coal or ashes, is certainly a great improvement. Add to this improved teeth and a better system of fitting, continuous gum is destined to become very popular. In taking impressions I invariably use plaster, as it represents the normal position of the parts; wax will push the soft tissues out of place. My system of studying the mouth and its conditions enables me to take a better impression than I could do otherwise, also to so change or modify the cast as to properly relieve the mouth from coming in uneven contact with a rigid denture. The success of fitting upper dentures is always less where the angles increase over 45°, the intermediate fluids cause the two surfaces to slip past each other. You can only estimate the retaining force according to the diameter of horizontal surface. In each case in making the proper relief for a denture it must cover four-fifths of the lingual surface and sometimes the dense portion of the maxillary bone. The more rigid the mouth, the less relief; the more spongy the alveolar ridge, the greater the center relief. Nearly every dentist who makes a set of rubber teeth, destroys the perfect contact by placing too much rubber, while packing on the central portion of the dental arch, so that when the flask is brought together the greatest pressure is brought to bear in the center of the palatine surface, causing the plaster to give way, thus producing a denture that will find its greatest contact in the center of the mouth, when it should be on the alveolar ridge. To avoid this danger, I always leave the center unpacked, and depend on the surplus rubber to fill it up.

*Dr. Taft.* What about plaster impressions; do you use plaster for the upper and lower jaw, both?

*Dr. Land.* Well I could name mouths where it would not be successful to use plaster, but I very seldom have to use wax; have not taken a wax impression of the upper portion of the mouth for fifteen years. Have taken impressions of the lower, because I often find the muscles so flabby that the plaster would not be stiff enough to push them out of the way. Then, there are mouths that would not promise a perfect success with plaster.

*Dr. Taft.* Do you wax the teeth?

*Dr. Land.* I do; and sometimes put a little lard on them, it being clean and almost tasteless; sometimes I put a little cotton batting between the teeth and around them.

*Dr. James.* How do you mix your plaster; thick, or thin?

*Dr. Land.* I take the happy medium. Am very careful, first, to fill up the mouth level with the alveolar ridge by placing the plaster there with a spatula, then put just enough in the impression cup to finish the work; press the cup up at the back first, and graduallly force the plaster forward, not having any excess of plaster, it can not run down the throat.

The retention of a denture is due especially to the adhesive attraction that all fluid substances have for solid bodies. Any denture that will support moisture equally distributed between it and the mouth, secures the best fit, and is a manifestation of capillary force. In regard to the denture fitting the mouth by close contact, it is not due to atmospheric pressure, nor can it be compared to the force that holds two pieces of polished glass together. My belief, in the case of two polished surfaces, is that they are brought just within the range of their molecular attraction. In proof of this theory, the pieces of glass will hold together in a vacuum. The adhesion induced by an intermediate fluid will also remain together, while in a vacuum, under the same condition. Atmospheric pressure ceases to exist. There can be no manifestation of atmospheric pressure without an air space and an exhausting means.

*Dr. H. A. Smith.* I am not sure whether it is cohesion or adhesion which operates most favorably upon the denture. I am inclined to think that the capillary force is what keeps the plate in proper position, and if that is true, the question arises whether a rough plate is not better than a smooth one. It occurs to me that very many plates are maintained by capillary attraction.

*Dr. Williams.* It is a very easy matter to demonstrate the fact that the force which holds two pieces of perfectly fitting glass to each other, is not the pressure of the atmosphere; that it is some other force than the pressure of the air, and it is readily demonstrated that it is an internal force of attraction inherent in the pieces of glass. Whether we should call that cohesive attraction or adhesive may be a question, but in my mind I should call it cohesive attraction. It is precisely the same force that binds the molecules in a solid body together, though not acting with the same force that it acts in a perfectly solid body, from the fact that the approach of the molecules of the two pieces of glass can never be made as perfect as in the natural solid body. We have been satisfied for a long time that this is the force upon which we should depend in securing the denture in position, and holding it in the mouth, and that we should not depend upon the pressure force. We all know how injurious dentures are to the tissues of the mouth. Nature abhors a vacuum. The air is exhausted from the cavity, and immediately the tissues of the mouth, so far as they are able, rush in to fill the vacuum, thus producing an abnormal condition of the mucous membrane of the roof of the mouth.

I depend solely upon what seems to me to be either cohesive or adhesive attraction. It is attractive force, whatever else we may say of it, and I think that attractive force is the force that should be employed in adjusting a plate in the mouth, and not air pressure.

*Dr. Harroun.* I had a chance to see how they work out in the

Western States. One old fellow told me that he had to use large air chambers to make the plate "go" the first time. Some of his work was very creditable to him. He found it necessary to extract a good bicuspid for a patient, and transplanted it into his own mouth, where it is doing well. After considerable observation, I find that the dentistry done in the Western States is not equal to that done here.

*Dr. H. H. Harrison.* I am glad to see there is some interest being taken in prosthetic dentistry, and I think there are advancements being made every day. In the first place, the discarding of the air chambers is an advance. Another thing, I think there is an advance in the use of single teeth in artificial dentures. I don't think we ought to discard rubber though, because I believe it occupies a good place in the profession, and while it may not be the best, still it is a substance that can be well used to advantage by the operator, and the persons for whom it is used.

*Dr. Taft.* There is one method by which rubber plates were made some years ago, which is better than any that has been employed since its introduction, and that is the gold web; you can use thinner plate, and then the metal will somewhat lessen the non-conducting power of the rubber. I saw simple rubber plate, not long since, that was made twenty-four years ago, and has been worn all these years. But these web plates are a decided improvement over the rubber alone, and seem to me to wear longer and do better than anything of the cheaper sort.

*Dr. Berry.* So many are needing artificial substitutes, that it is best that we should avail ourselves of all practical improvements, and all of the knowledge that we can get. Doctor Allen taught us that every case should be studied, and I think that is right.

Now, you can't make a set of teeth in a hurry; to make them right requires time. There are several other little matters which have been brought out, which I regard as important. To pay attention to them would obviate much difficulty with patients.

Dr. Taft spoke of the gold web we used formerly. I regarded it then, and do now, as a very good thing. We used to buy that in the market, and whenever we were not satisfied with that, we had an arrangement by which we could get that web as we wanted it from the manufacturers. We had the wire woven by the Bromwell Co., and it was a very good thing, but one of the drawbacks was, the patients expected too much. It was thinner and lighter, but then they got it in their heads that because you improved the plate the teeth ought not to break.

*Dr. Rehwinkel.* Some allusions have been made this evening to the soreness of the mouth under gold and rubber plates. Above eight years ago I made a partial gold plate for a gentleman, and when he afterwards came back to have some teeth filled, I found that the whole roof of the mouth and the tissues some distance back in the throat were in a high state of inflammation. This condition of things was produced by his habit of wearing the plate day and night. I advised him to leave it out at intervals, and not to wear it at night; the result was, a great improvement of the parts. I am of the opinion that hundreds of dentists advise their patients to wear their plates day and night. This is *wrong*, for nine cases out of ten of inflammation of the soft parts of the mouth are caused by the continual pressure of the plate in the mouth.

I have had cases in which the mouth would not tolerate rubber, so I took to lining the plate with gold. This, I soon found, cost a great deal of time and labor on account of its difficulty. I finally hit upon a very simple plan: Roll out coin gold very thin; trim and swadge it to the model, and around the edges punch holes and dovetails. The plate of gold when imbedded and vulcanized in the rubber, can not be withdrawn.

*Dr. H. A. Smith.* I have occasionally used Williams' surface gold to obviate the trouble spoken of by Dr. Rehwinkle. In attaching any metal to rubber plates, a somewhat roughened surface is preferable to a smooth one. I have seen many beautiful plates, made by Dr. Taft, with his woven gold, but plates so made always fracture on a strain. Again, if such a plate be reduced too thin it looses strength, and the method was abandoned on that account.

*Dr. Berry.* You may leave as much rubber as you want in the front part of the plate, but the back must be made thin.

*Dr. H. A. Smith.* Rubber alone can be trimmed that way, but the web plates were reduced so thin that no advantage was had by the use of it.

*Dr. Taft.* Some of the strongest plates I ever made contained the gold web. I never cut the plate down to the bare thickness of the web. In the middle and back the plate can be reduced in thickness, as Dr. Berry says. Gauge the quantity of rubber to suit the case. An extra strong plate can be made by the addition of a bar of gold. So far as fractures are concerned, I have, as every one else has, seen rubber plates split from end to end.

Adjourned.

SECOND DAY.—MORNING SESSION.

The meeting was called to order by President Berry, at 9:30; and minutes of previous meeting read and approved. The discussion of "Improvements in Artificial Dentures" was resumed.

*Dr. Callahan.* I hope that remarks upon this subject will be made by the older and more experienced members, in order to lend the weight of their influence to stimulate Ohio dentists to take more interest in continuous gum work, as I believe our improvement lies in that direction. Very many do not engage in it because they dread the furnace work. Dr. Land certainly knows something about the improvements being made in the construction of furnaces, and I should very much like to hear from him.

*Dr. Land.* The difference between the coal and the gas furnace is in the convenience to the dentist. The old method was a fifteen hours' job of heat, dirt, and vexation. With a gas furnace, a dentist who is in the practice of both branches of the profession can bake a piece of continuous gum in several heats of from fifteen to twenty minutes duration, and that at such times and intervals that will not conflict with his engagements at the chair. The gas furnace is always sure, reliable, and easy of handling, while on the other hand, a coal or coke furnace does not always work right. Dr. Allen's body for continuous gum is the most reliable and gives the best artistic results. It is hard to make any improvement over Allen's method, except, possibly, to so change the position of the pins that his teeth can be utilized in other methods, and to improve articulation.

The system of reliefs in preparing the plate will give success in the large majority of cases, as the mouth will almost always adapt itself to the denture.

I procured a Verrier furnace, and must say that I was delighted with it, but found that it would "gas." The desirable point is to secure perfect combustion. An improved muffle and fire-clay, both properly managed, will aid very much in securing this.

It is almost impossible to produce a perfect combustion of the hydro-carbon; the result of the combustion is carbonic acid gas and water. An excess of oxygen is desirable, only it is not possible. The reason of the blistering of a piece is that the hydro-carbon gas gets into the piece during the first biscuiting. During the enamelling the heat is much higher, and the gas trying to get out from under the semi-fluid mass of enamel fails, but leaves the bulge we call a blister. The cracking and checking of continuous gum is due to a "burning" of the piece during the second biscuiting; it is much better to have a "raw" second biscuiting, than that the first should be overdone.

I have made some experiments which, however, are not complete as yet, regarding the blistering of pieces. I have found, so far, that pure oxygen is almost inert, and only acts in the presence of hydrogen. The hydro-carbon gas is the immediate cause of the blistering.

*Dr. James.* This subject is at this time of great importance to us. It seems to me that for some years we have been advancing backwards in this branch of our profession. We would do well to begin again with first principles—go back to gold and continuous gum, and learn to work them as they used to be worked in their perfection and beauty.

Rubber and celluloid have their place, no doubt, but there are few instances in which gold and continuous gum can not be used with the greatest success. In those cases where there is cracking in the first biscuiting of a continuous gum piece, or where there is a slight warp, or spring of the plate, it may be put on the die and pressed back to place, even at the risk of its cracking somewhere, for this can be remedied by filling up with new body and re-biscuiting.

If the gas furnace can be so perfected as to do away with the liability of blistering, it certainly will simplify the work to be done, besides enabling many to procure furnaces who otherwise could not afford the great expense of the furnaces formerly in use.

*Dr. Berry.* I very well remember the time when a dentist was of necessity obliged to devote a whole day at the furnace, consequently keeping him away from his chair.

If he deferred the burning till after night, it not only deprived him of time that should be devoted to rest, but it had a most injurious effect upon his eyes. Continuous gum is, no doubt, a most desirable thing in the way of a substitute for the natural organs, but it need not always be the larger part of a man's personal being, as I once found. Dr. Allen made a piece for a patient, and it weighed at least half a pound. [Laughter.] No, I am speaking in all seriousness. That gentleman possessed no piece of property that he valued more highly, or was more proud of, than that set of teeth. And this will always be the case with patients for whom you make a fine and artistic piece of work; it will repay you, and the patient as well.

An artistic dentist is every bit as much an artist as he who paints beautiful pictures of the face, upon canvas, for we restore the lost beauty of outline, feature, and expression.

*Dr. James.* In my remarks a short while ago, I forgot to call attention to cast plates; cheoplasty can, and does, offer the facility of making artistic work that is both satisfactory to the patient and the dentist who perseveringly toils to imitate nature, and this without incurring the expense that is a bar to many who would otherwise enjoy the highest fruits of our skill.

*Dr. Harroun.* A case came under my observation some time ago, in which the patient had had the process cut away by some one. There was much loss of tissue, due both to the cutting away and subsequent absorption. This, of course, complicated matters, and the patient, with an eye to economy, tried several of the cheap fellows, but without success, so far as he was concerned. I made a piece for him on Watt's metal which was a perfect success in every way, filling all the requirements, much to the delight of the patient, and to my own satisfaction. We can obviate many difficulties if we will give our minds to the work, and get down to it with a will. We all can work continuous gum successfully, if we are determined to do so.

*Dr. Callahan.* I have often noticed that a continuous gum piece shrinks, or contracts, at the heels after it has been biscuited, and I would like Dr. Land to be kind enough to tell us what he does in such instances.

*Dr. Land.* The shrinkage about which Dr. Callahan has just inquired, both above and below, is always from the outside toward the inside; if we make a study of each case that is presented experience will guide us as to how much allowance to make in order to counteract it.

The best way to spring a plate into its place again is from the middle. You can spread a plate by making it go against the sides, but you can not draw it in. Neither can a plate be altered if it has sprung, unless provision has been made for such a contingency by studying the angles of the mouth to determine the amount of allowance to be made.

On motion, the subject was passed.

Dr. Harrison, of the Committee on Voluntary Essays, said that he had an historical poem written by Dr. Siddall, of Oberlin; it was accepted and read.

Dr. Robinson exhibited some models showing the effects of hereditary syphilis upon the teeth. He had examined the mouths of 400 deaf mutes at the institution at Columbus, and out of that number found thirty-one cases of hereditary syphilis, all showing more or less marked effects npon the teeth.

Dr. James moved a suspension of the rules in order to offer a resolution relating to the dental law. (We failed to get a copy of that resolution.—Rep.)

*Dr. Butler.* I am very much in favor of a registry law, as in those States where there is such a law, it has done away with the State Examining Board.

*Dr. Watt.* We are only wasting time in discussing this matter, neither are we instructing the committee whose duty it is to attend to it; resolution carried.

*Dr. Rehwinkel.* I will take advantage of this moment to say to the Society that, during last night, I received from various members of this and foreign countries some letters in regard to persons not qualified to practice dentistry. Dr. Gray, of Zanesville, who knows more about the matter than anybody else, states that in the southern part of the State there are a number of persons practicing dentistry under a diploma from Delavan; that this institution is selling diplomas for $12, and that it is nothing but a fraud throughout. This college is not recognized by any Board of Examiners, or anybody else, but, unfortunately, our law is so lame that we can do nothing. There is a good deal of illegal practice done in the State, and it is nobody's business to enter into a prosecution. The truth is, our present law is in such a condition, that it is exceedingly uncertain whether a successful prosecution could be entered into and carried through, but it seems that these diplomas are not good, and ought not to be allowed to come in here.

Any college which does not act up to the full requirements of the established standard ought not to be recognized. Any college which does not require a two years course ought not to be regarded as a square institution. Of course, a term in a medical college is equivalent to one course in a dental college; but that institution in Delavan requires no course whatever, and the profession ought to take some action in the matter. I move that the subject be the special order of business at 2 o'clock, and that we now adjourn. Carried.

SECOND DAY—P. M. SESSION.

The meeting was called to order by President Berry at 2:30. The Secretary read what purported to be minutes of the morning session. Dr. Watt said as they were not complete. A motion was carried to dispense with the reading of them. The Chair appointed Drs. Rehwinkel, James, and Williams on the Committee on Dental Law, which appears to be the intent of Dr. James' resolution. Special order of business was then taken up.

*Dr. Taft.* I hardly know what to say in reference to this subject, and what action the Society can take in the matter, I am not fully decided. The fact that this bogus institution in Wisconsin has been in operation for years is familiar to everybody. So flagrant is it, and so extensive have been its operations that it has brought great reproach upon all the institutions of this country. It has been mainly on account of this that other countries have disregarded American diplomas, and the question, of course, comes home to every member of the profession. Every one has an interest in this matter, and should have; and if anything can be done by which this evil can be remedied, or by which the power of that institution could be controlled, or broken, it ought to be done.

It seems that the State of Ohio might assist in presenting the true aspect of affairs to the Legislature of Wisconsin, and ask that the institution be repressed. If a memorial could be sent from this State, it might have some influence.

If the different State Societies would each make an effort, or if they would send strong protests to the Legislature of Wisconsin, it would have great influence. It is for us to contribute any effort we can to the accomplishment of this result, and I think we are all prohibitionists so far as that institution is concerned. It has wrought mischief, not only by sending out bogus diplomas, but has brought into disrepute the profession of this country.

*Dr. C. R. Butler.* If our Society disapproves of such conduct, something ought to be done to show its disapproval. Diplomas from colleges in this country are not received with that respect to which they are entitled, and particularly is this true in Germany, and if there is anything that can be done to remedy this evil, we ought to do it.

*Dr. Watt.* I don't know anything the Society can do, but there is something radically wrong. Dr. N. W. Williams, my friend, states it is very common for Germans to come to this country, spend part of a season here, buy a diploma, and then go back and compete with our American dentists abroad. Thus the term "American Dentist," instead of being an honor, becomes a reproach.

*Dr. Smith.* There is a letter here from Dr. G. R. Richter referring to the matter and speaking in regard to having its charter taken away; if we can bring any influence upon the State which will secure this result, it is a very proper thing to do.

*Dr. Keely.* About four years ago I had the pleasure of attending the State Society at Milwaukee, and the head of this college and one of the professors came into the Society's rooms. I don't think I ever saw such a commotion as their appearance gave rise to in my life. I was told that the head professor had been a medical man of good standing and had been very prominent in the State, and his brother had used his influence to prevent his getting into the college, but he went into it believing there was a pile of money in it. I had a good deal of sympathy for him, but none for the college, and I say this to show the feeling that exists in regard to the college in Wisconsin.

Dr. Smith then read the letter from Dr. Richter, previously referred to.

*Dr. Rehwinkel.* We missed the best opportunity we ever had, when the druggists passed their law which embodies all the features necessary for us. The time has now come when something must be done, either enforce the law or we shall have to turn quacks in self defense.

The following resolution was offered by Dr. Taft:

WHEREAS, It appears that certain parties are practicing dentistry contrary to the laws of the State of Ohio, and screening themselves under a diploma issued by the Wisconsin Dental College, at Delavan, in that State, said diploma not being recognized by any State society in this country:—

*Resolved*, That this Society greatly deplores the existence and mode of procedure employed by the said college; which issues diplomas entirely irrespective of qualifications and without any instruction pertaining to dentistry.

And we pledge the Wisconsin Dental Society any aid in our power for the suppression of this quack institution.

The resolution was carried without an objection.

*Dr. Callahan.*—As many members are about leaving, I move that the rules be suspended and that we proceed to the election of officers for the ensuing year; carried—the result being as follows: President, C. H. James, Cincinnati; Vice-President, H. H. Harrison, Cadiz; Secretary, J. R. Callahan, Hillsboro; Treasurer, G. W. Keely, Oxford. Dr. H. A. Smith was re-elected as a member of the State Dental Examining Board.

A motion was then carried to install the officers elect, the Chair appointing Drs. Rehwinkel and Taft to perform that ceremony.

All the subjects on the programme, 1st, 2nd, 3rd, 4th, and 5th were passed, and the 6th, "Food, Digestion, and Nutrition in Relation to the Healthful Condition of the Teeth and Fluids of the Mouth," was taken up.

Dr. Rehwinkel read a paper bearing on the subject, written by Dr. Lilly, of Circleville. The paper was not discussed as time pressed and other business had to be attended to. Dr. Rehwinkel also made a report for the State Examining Board; two candidates having presented themselves, and passing a satisfactory examination, were granted license to practice.

The committee appointed to nominate delegates to the American Dental Association reported the following list: Drs. J. R. Callahan, E. G. Betty, H. F. Harvey, W. H. Todd, J. H. Warner, C. H. James, J. E. Robinson, and A. O. Howe.

The committee appointed to examine into the merits of the Horton case made the following report:

The committee appointed to consider the matter in controversy between Dr. W. P. Horton and the Publication Committee, relating to the efforts of the former to obtain posession of papers read before the society at the last annual meeting, in advance of their appearance in the transactions of the Society, would respectfully report, that they have carefully considered the statements and evidence presented by both sides, and are of the opinion that Dr. Horton, as a member, has not violated any written law of the Society; yet, in what appears to the committee, in his over-zeal in the capacity of a reporter for a dental publication to obtain possession of papers for use, has not observed the rule which he admits has obtained in this Society, viz.: that all papers read and accepted become the property of the Society, and can not be published in any paper or journal other than the Transactions, without the consent of the Society, and that he is clearly chargeable with discourtesy towards this body.

H. A. SMITH,
A. F. EMMENGER,
F. A. HUNTER.

Drs. Horton and Warner wished to discuss the report at length, but were stopped by the report being adopted as read, as the committee had been empowered to investigate the charges, and make a final report. Dr. Taft then offered this resolution in writing:

*Resolved*, That upon the final adjournment of this session, the Society adjourn *sine die*, and that at that time the organization be dissolved.

This was accepted and adopted by an overwhelming vote. Dr. Harrison then offered the following:

*Resolved*, That if from lack of funds, or any other cause, a volume of Transactions be not issued, the papers read at this meeting be regarded as the joint property of the DENTAL REGISTER, and the *Ohio State Journal of Dental Science.*

Dr. Horton very zealously wanted the *Independent Practitioner* to have a share of the spoils, but his motion to that effect was voted down, and the resolution unanimously adopted.

Dr. J. H. Warner, of the Publication Committee, reported the publication of the Transactions for 1883, in which he showed that the amount received for advertisements exceeded the cost of publication by over one hundred dollars. His report was accepted and adopted. A motion was then carried to pay over to him the sum of $100.00 in acknowledgement of his services in getting out the Transactions.

His salary of $25.00 as Secretary was then voted and paid to him. He then presented a bill of repairs amounting to some seventy-five cents, for engraving the name of the last President upon the gavel. There still being a small sum remaining in the treasury (though an assessment was suggested, under the impression that the treasury was cleaned out), the bill was duly audited, allowed, and the usual warrant drawn upon the treasury, and the debt liquidated, much to the relief of those interested in clearing away all the debts incurred by the Society.

The Society then adjourned *sine die*, although it was a pretty lively corpse, as the proceedings published in the last number of the REGISTER give ample evidence.

